# Successful laparoscopic repair for reduction en masse of infantile inguinal hernia: a case report of this rare condition

**DOI:** 10.1186/s40792-022-01535-1

**Published:** 2022-09-26

**Authors:** Keisuke Yano, Koshiro Sugita, Koji Yamada, Mayu Matsui, Waka Yamada, Chihiro Kedoin, Masakazu Murakami, Toshio Harumatsu, Shun Onishi, Takafumi Kawano, Mitsuru Muto, Satoshi Ieiri

**Affiliations:** 1 Department of Pediatric Surgery, Research Field in Medicine and Health Sciences, Medical and Dental Sciences Area, Research and Education Assembly, Kagoshima University, 8-35-1 Sakuragaoka, Kagoshima City, 890-8520 Japan; 2 Clinical Training Center, Kagoshima University Hospital, Kagoshima, Japan

**Keywords:** Reduction en masse, Laparoscopic percutaneous extraperitoneal closure, Pediatric surgery, Inguinal hernia, Incarcerated hernia, Small bowel obstruction, Manual reduction, Rare condition

## Abstract

**Background:**

Reduction en masse (REM) is a rare condition following manual inguinal hernia (IH) reduction in which a hernia sac is reduced back into the preperitoneal space with a loop of the bowel incarcerated at the neck of the sac. It resembles successful manual reduction and may thus be overlooked easily. We herein report an infantile case of REM of an IH that was successfully treated laparoscopically.

**Case presentation:**

A 10-month-old boy with a surgical history of bilateral open IH repair at 4 months old presented with a bulge in his left groin and vomiting. A left incarcerated recurrent IH was suspected, and manual reduction was performed. The hernia was apparently reduced successfully, but abdominal distention and vomiting persisted. He was admitted for further observation due to the symptoms. On day 2 after admission, abdominal X-ray showed extensive small bowel obstruction (SBO). Enhanced computed tomography (CT) revealed protrusion of the small bowel with a closed-loop in the left groin. A closed-loop SBO due to postoperative adhesion or an internal hernia was suspected. To assess the etiology of SBO, emergent laparoscopic exploration with hernia repair was planned. Laparoscopy revealed REM of the left incarcerated IH with a thickened peritoneum at the neck of the sac. Laparoscopic reduction was performed, and the incarcerated small bowel showed no signs of ischemia. The hernia sac was not associated with the previously ligated processes vaginalis, which had been closed by a previous Potts’ procedure. It was located at the inside of the processes vaginalis. The sac was successfully closed by laparoscopic percutaneous extraperitoneal closure procedures, and iliopubic tract repair was also performed via the previous inguinal incision. The postoperative course was uneventful.

**Conclusion:**

Pediatric IH is due to the patent processes vaginalis, and REM is extremely rare. Laparoscopic surgery for REM is a relatively common and useful approach for the diagnosis and treatment of adults. In our infantile case, the laparoscopic approach was similarly effective for both investigating the cause of SBO and performing high ligation of the sac for this rare condition with IH.

**Supplementary Information:**

The online version contains supplementary material available at 10.1186/s40792-022-01535-1.

## Background

Reduction en masse (REM) is a rare condition following manual inguinal hernia (IH) reduction in which a hernia sac is reduced back into the preperitoneal space with a loop of the bowel incarcerated at the neck of the sac [1]. It is necessary to perform emergent surgical treatment for REM because incarcerated hernia remains. However, the diagnosis of REM is often difficult, as it is a rare complication, and it initially appears as if manual reduction was successfully performed. Therefore, when it occurs, it is easily overlooked, which results in a delayed diagnosis and the need for emergent surgery.

We herein report an infantile case of REM of IH that was successfully treated by laparoscopic procedures.

## Case presentation

**Fig. 1:**
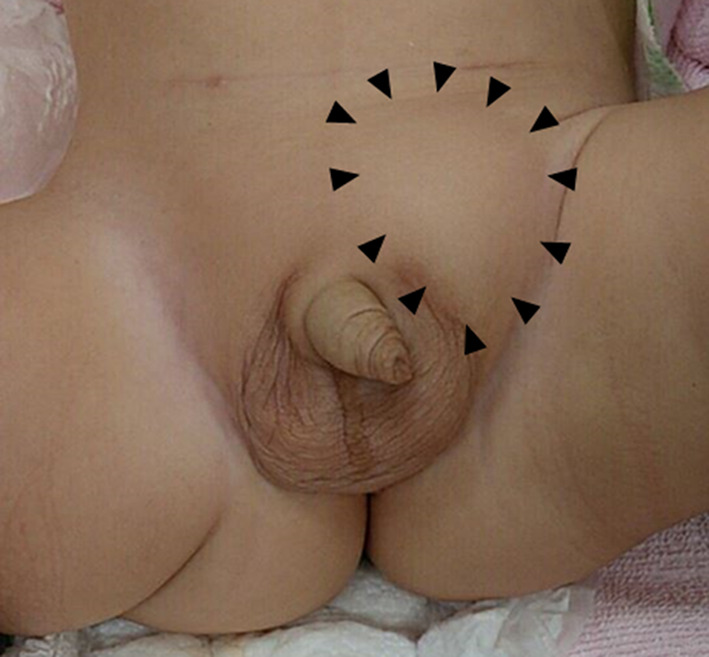
A bulge at the patient’s left groin

The case was a 10-month-old boy who had a surgical history of bilateral open inguinal hernia repair at 4 months of age at our institution. He presented with a bulge on his left groin and vomiting (Fig. 1). A left incarcerated recurrent inguinal hernia was suspected, and manual reduction was performed. The hernia was apparently reduced successfully, but abdominal distention and vomiting persisted. Therefore, he was admitted for further observation due to the symptoms.

**Fig. 2:**
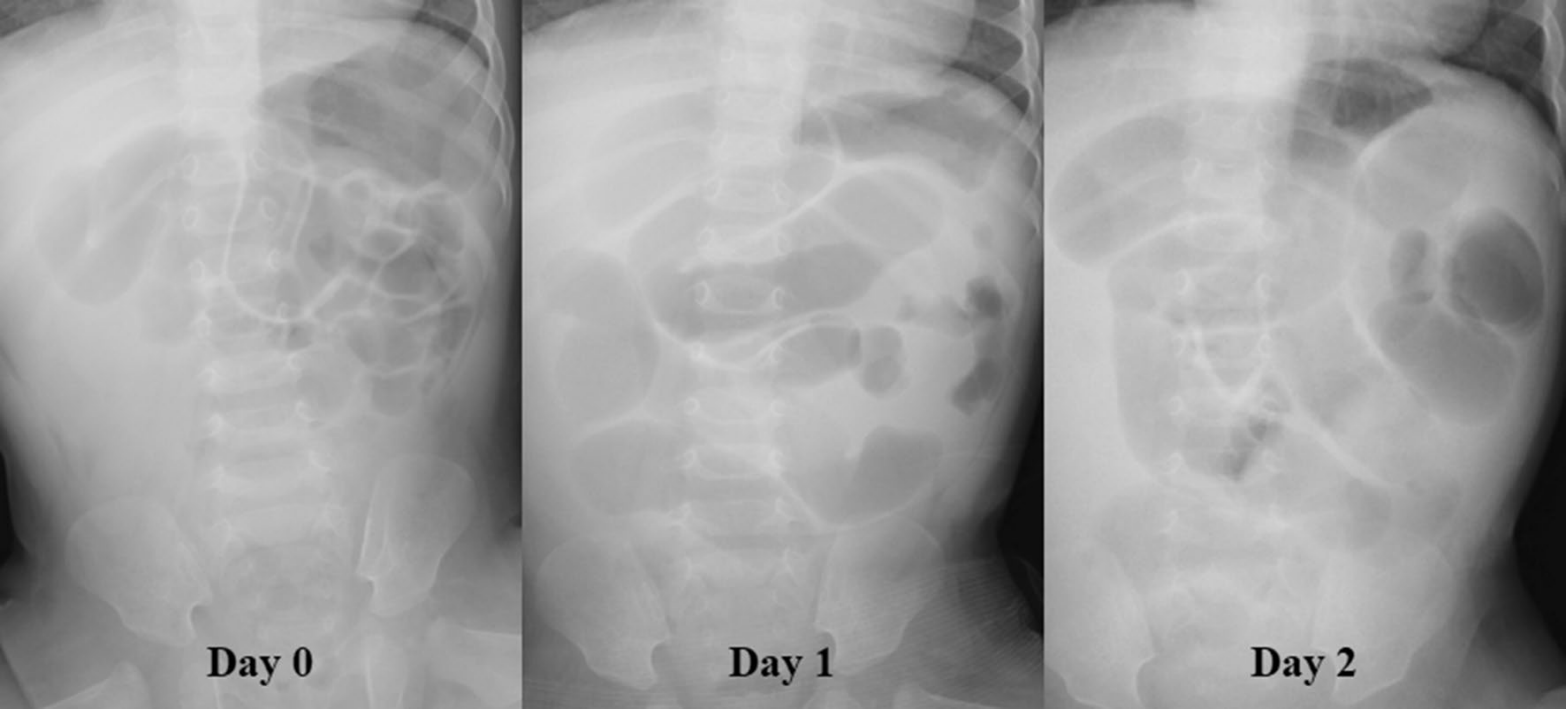
Abdominal X-ray findings

**Fig. 3:**
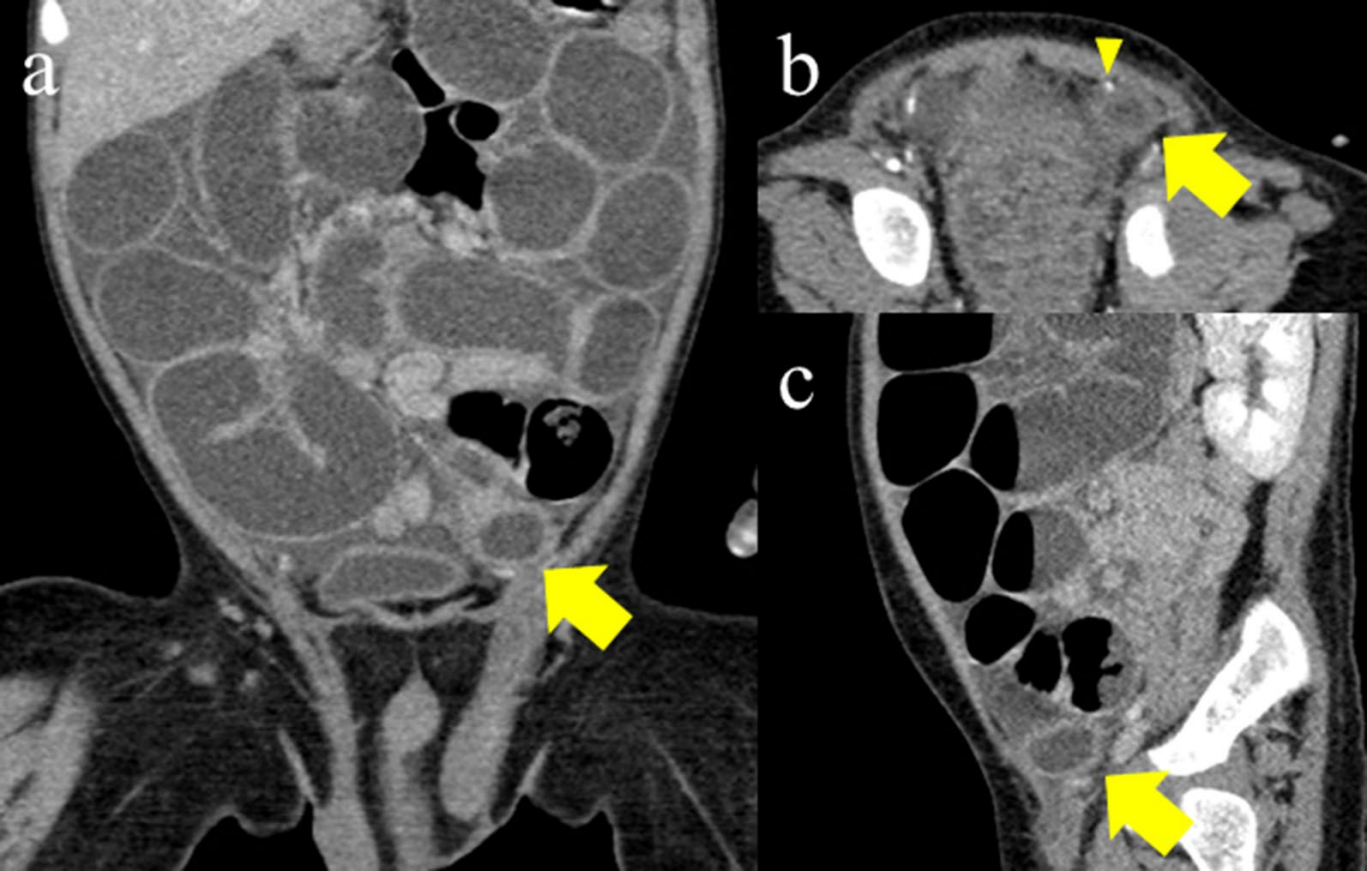
Enhanced CT findings. Closed loop in the left groin (yellow arrow). **a** Coronal section. **b** Axial section and inferior gastric artery (yellow arrowhead). **c** Sagittal section

Immediately after manual reduction and on the first day after admission, abdominal X-ray showed mild small bowel dilatation. On the second day after admission, abdominal X-ray showed extensive dilatation of the small bowel and a paucity of gas in the colon (Fig. 2). Enhanced computed tomography (CT) was performed to investigate causes of small bowel obstruction (SBO). It revealed protrusion of the small bowel with a closed-loop in the left groin (Fig. 3). SBO with a closed-loop due to postoperative adhesion or internal hernia was suspected. To clarify the cause of the SBO, emergent laparoscopic exploration with recurrent inguinal hernia repair was planned.

**Fig. 4:**
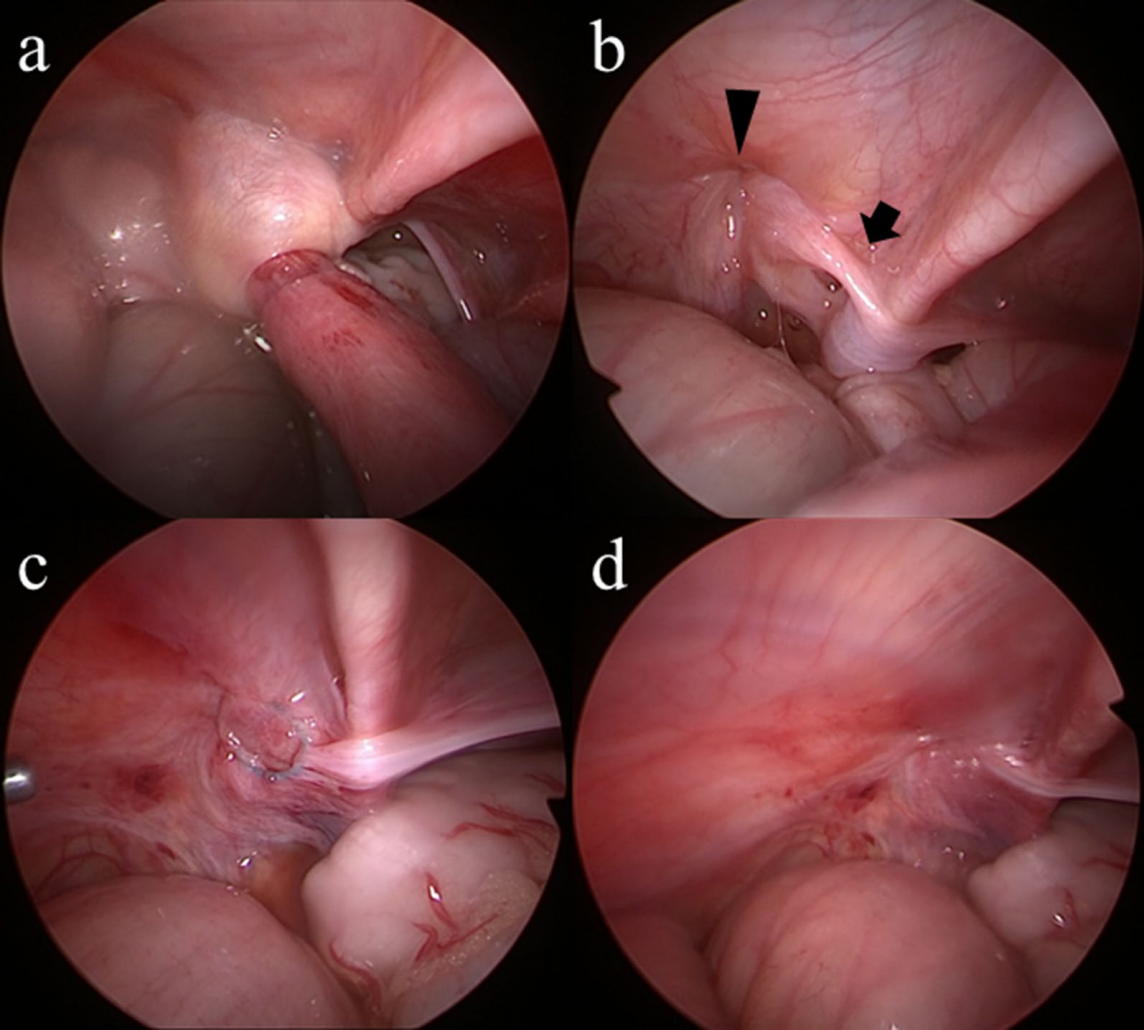
Operative findings. **a** Small bowel dilatation and a loop of small intestine remained trapped in the hernia with a thickened peritoneum at the neck of the sac in the left inguinal. **b** The hernia sac(black arrow) was not associated with the processus vaginalis(black arrowhead), which had been closed by a previous open procedure. **c** The hernia neck was encircled without skipping. **d** The hernia neck received high ligation

Under general anesthesia with the patient in a supine position, the umbilicus was opened through a 1.5-cm longitudinal incision, and a wound retractor (LAP PROTECTOR Mini-mini; Hakko Co., Ltd., Tokyo, Japan) was applied. Two 5-mm trocars (scope and operator’s right hand, respectively) were inserted through a multichannel port device (E・Z access; Hakko Co., Ltd.) to attach to the wound retractor, and pneumoperitoneum was established (8 mm Hg, 5 L/min CO_2_ insufflation). Laparoscopy revealed a small amount of ascites, small bowel dilatation and a loop of small remained trapped in the hernia with a thickened peritoneum at the neck of the sac in the left inguinal lesion (Fig. 4a). Laparoscopic reduction was performed by grasping and pulling the incarcerated small intestine using an atraumatic grasper. After successful reduction, the incarcerated small bowel showed no signs of ischemia, so intestinal anastomosis was not performed. Laparoscopy and inserting forceps revealed that the processus vaginalis (PV) had remained closed by a previous operation for IH. The hernia sac at the laparoscopic procedure was not associated with the PV that had been closed by previous open IH repair. It was also located away from the testicular artery and vein as well as the vas deferens, and not inside of the inferior gastric artery (Fig. 4b). To ligate the neck of the hernia sac, a Lapa-Her-Closure needle™ (Hakko Co., Ltd.) was percutaneously inserted into the preperitoneal space just above the neck of the sac. The needle came down halfway around the neck, passed through the peritoneum, released a suture, and then went back, this time coming through the opposite side halfway around the neck and catching the suture before exiting the body (Fig. 4c). The neck was encircled without skipping, and the thickened peritoneum was high ligated (Fig. 4d). Because direct hernia was also suspected, we additionally performed iliopubic tract repair via the previous inguinal incision. The postoperative course was uneventful (Additional file 1).

## Discussion

The incidence of REM is reportedly 1 in 13,000 hernias [2]; however, because of an increasing number of early-period surgical repair procedures for hernias after a definitive diagnosis, the incidence is likely far less than that reported[3]. The hernia sack is made by persistent stimulation of the peritoneum, which results in fibrotic thickening and stenosis, leading to the formation of a new hernia phylum [4]. Despite the disappearance of groin swelling, some patients complain of abdominal symptoms and prolonged findings of SBO [5]. When SBO is found after manual reduction of IH, as in the present case, it is possible that REM has occurred, so caution is required.

The occurrence of REM requires several criteria be met: (1) the hernia sac must have a narrow neck, making it difficult for the bowel to move back out of the sac; (2) the hernia sac must be mobile within the inguinal canal; and (3) there must be enough laxity in the peritoneal cavity without displacing the bowel loops from the sac [6]. In some cases, REM can be manually reduced by the patients themselves [7]. It is generally thought that children are at a lower risk of REM than adults because their hernia sacs tend to adhere tightly to the neighboring cord structures [8]. Regarding REM in pediatric patients, there are two case reports available [8, 9]. Both cases had findings of SBO after manual reduction of IH, similar to our present case. On the other hand, not similar to our present case, they were not recurrent IH after surgery and older than our present case. Their more detailed type of IH was not described, however, it was considered that lack of adhere and vulnerable of their IH sac caused REM [8, 9]. In our present case, it was considered that the initial Potts procedure made the IH sac lacking adhere and vulnerable similarly to them.

Regarding the mechanism underlying recurrent IH after the Potts procedure at four months old in our present case, it was not associated with the recurrence of patent PV. The laparoscopic findings showed the incarcerated small bowel located inside the previously ligated PV. Retrospectivity, enhanced CT revealed that hernia sac and trapped small bowel had not been inside of the inferior gastric artery, and they appeared that they had headed into internal inguinal ring through outside of the inferior gastric artery (Fig. 3). The mechanism underlying recurrent IH in our present case is thus considered to not involve the recurrence of repaired indirect hernia or complicated congenital direct hernia after the Potts procedure. Our present case is thought be a recurrence as de novo type of external IH. There have been reports concerning the effectiveness of laparoscopic reduction for REM of IH in adult cases [10]. Similarly, in our present case, a laparoscopic procedure was also effective for the treatment and diagnosis of this rare condition of pediatric REM and recurrent IH.

## Conclusion

In cases of SBO after manual reduction of IH, the potential involvement of REM should be considered. Laparoscopic procedures for REM are effective for the diagnosis and treatment of REM in infantile cases of this extremely rare condition.

## Supplementary Information

**Additional file 1.**

## Data Availability

The datasets supporting the conclusions of this article are included within the article.

## Abbreviations

REM: Reduction en masse
IH: Inguinal hernia
SBO: Short bowel obstruction
PV: Processus vaginalis

## References

[1] Ravikumar H, Babu S, Govindrajan M, Kalyanpur A (2009). Reduction en-masse of inguinal hernia with strangulated obstruction. Biomed Imaging Interv J.

[2] He P (1931). Strangulated hernia reduced en masse. Surg Gynecol Obstet.

[3] Brady MP, Veith FJ (1964). Reduction en masse of incarcerated inguinal hernia. A new look at an old problem. Am J Surg.

[4] Oshidari B, Ebrahimian M, Nakhaei M, Ghayebi N (2022). Laparoscopic relief of reduction en-masse in an inguinal hernia: a case report. Int J Surg Case Rep.

[5] Sahoo MR, Kumar A. Laparosopic management of reduction-en-masse. BMJ Case Rep. 2012;2012. 10.1136/bcr-2012-007919 PMC4545101 23264162

[6] Nason LH, Mixter CG (1935). Hernia reduced en masse. J Am Med Assoc.

[7] Mings H, Olson JD (1965). Reduction "en masse" of Groin Herniae. Arch Surg.

[8] Olguner M, Ağartan C, Akgür FM, Aktuğ T (2000). Pediatric case of hernia reduction en masse. Pediatr Int.

[9] Bernie AM, Schwanke T, Keutgen X, Spigland N (2012). Reduction en masse in a 7-year-old boy: an interesting case. J Pediatr Surg.

[10] Cao Y, Kohga A, Kawabe A, Yajima K, Okumura T, Yamashita K (2019). Case of reduction en masse who presented with no symptoms. Asian J Endosc Surg.

